# Is cardiac CT a reliable alternative for cardiac CMR in adult patients with a systemic right ventricle?

**DOI:** 10.1186/1532-429X-14-S1-P111

**Published:** 2012-02-01

**Authors:** Michiel M Winter, Soha Romeih, Maarten Groenink, Anje M Spijkerboer, Nico A Blom, Barbara J Mulder

**Affiliations:** 1 Cardiology, Academic Medical Center, Amsterdam, Netherlands; 2 Radiology, Academic Medical Center, Amsterdam, Netherlands; 3 Pediatric Cardiology, Academic Medical Center, Amsterdam, Netherlands

## Summary

Our manuscript addresses the reliability of cardiac CT as an alternative for cardiac MRI in patients with a systemic right ventricle. Currently, cardiac MRI is considered the gold standard for volumes and function measurements of the systemic right ventricle. However, 20% of patients with a systemic right ventricle is pacemaker dependent, and therefore unsuitable to undergo MRI. To our knowledge, this is the first study to evaluate whether cardiac CT provides a reliable alternative for volumes and function measurements of the systemic RV, which is morphologically very different from the subpulmonary RV. We found that cardiac CT provides a reliable alternative for cardiac MRI for volumes and function measurements of the systemic RV, although larger variability between measurements should be taken into account. However, we recommend restrictive patient selection, to avoid unnecessary exposure to radiation and contrast agents.

## Background

Cardiovascular Magnetic Resonance (CMR) imaging is considered the gold standard for volumes and function measurements of the systemic right ventricle (RV). However, 20% of patients with a systemic RV is pacemaker dependent, and unsuitable to undergo CMR. Multidetector Row Computed Tomography (MDCT) could provide a reliable alternative for CMR in these patients. The aim of this study was to compare variability of MDCT with CMR measurements.

## Methods

Thirty-five patients (23 with an atrially switched transposition of the great arteries, and 12 with a congenitally corrected transposition of the great arteries) underwent MDCT (n = 15; 47% male; 32 ± 8 yrs), or CMR (n = 20; 80% male; 35 ± 12 yrs). Systemic RV end diastolic volume, end systolic volume, stroke volume, and ejection fraction were obtained.RV evaluation was done 3 times by 2 independent observers (MW, SR with 2 and 4 years of experience in CMR and MDCT of adult patients with congenital heart diseases, respectively). The first observer analyzed all scans twice, with a minimal interval of 2 weeks between the first and second scan analysis, and blinded to the previous results. The second observer analyzed the scans once, blinded to the results of the first observer. The intra- and inter-observer variability for both modalities were assessed and compared.

## Results

**Figure 1:**
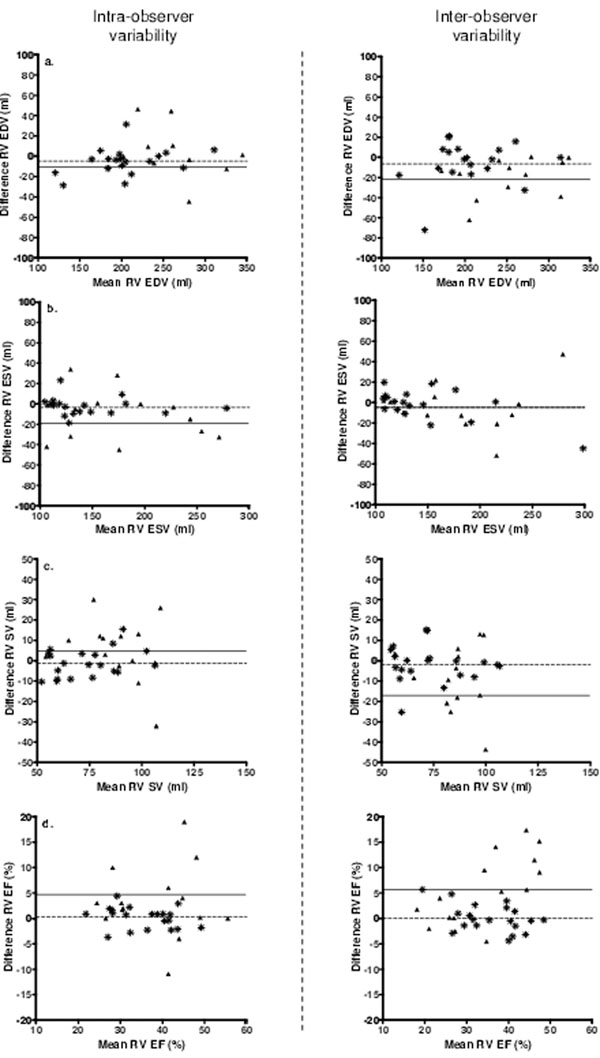
Bland-Altman plots depicting the intra- and interobserver variability between Multidetector Row Computed Tomography, and Cardiovascular Magnetic Resonance. Caption: Bland-Altman plots demonstrating the intra-observer (left side), and inter-observer (right side) variability of right ventricular a). end diastolic volume, b). end systolic volume, c). stroke volume, and d). ejection fraction. On the X-axis the mean value of both measurements, and on the Y-axis the difference between measurements. The Δ represent measurements performed with MDCT, the represents the mean of the differences between MDCT measurements. The represent measurements performed with CMR, the represent the mean of the differences between CMR measurements.

The Intra- and inter-observer variability data are summarized in Table 1. We found the intra-, and the inter-observer variability of volumes and function measurements of the systemic RV obtained with MDCT to be higher compared to those obtained with CMR. However, these differences in variability did not reach statistical significance, the only exception being the inter-observer variability of the systemic right ventricular stroke volume (12% with CMR vs. 32% with MDCT; p < 0.01, Figure 1).

**Table 1:** Intra- and inter-observer variability of measurements

Intra-observer variability											
	CMR (n=20)					MDCT (n=15)					p-value

Parameter	Average	Difference			CV	Average	Difference			CV	

EDV (ml)	212	-5	±	13	6%	294	-11	±	36	12%	N.S
ESV (ml)	139	-4	±	9	7%	200	-19	±	35	18%	N.S
SV (ml)	74	-1	±	7	9%	96	5	±	15	16%	N.S
EF (%)	36	0.1	±	2	6%	35	6	±	9	25%	N.S

Inter-observer variability											

	CMR (n=20)					MDCT (n=15)					p-value

Parameter	Average	Difference			CV	Average	Difference			CV	

EDV (ml)	213	-7	±	21	10%	277	-22	±	34	12%	N.S
ESV (ml)	139	-5	±	18	13%	189	-5	±	22	12%	N.S
SV (ml)	74	-2	±	9	12%	89	-17	±	29	32%	<0.01
EF (%)	36	-0.1	±	3	8%	35	-6	±	7	20%	NS

## Conclusions

MDCT provides a reliable alternative for CMR for volumes and function assessment in patients with a systemic RV, although larger variability between measurements should be taken into account. However, patient selection should be restrictive, to avoid unnecessary exposure to radiation and contrast agents.

## Funding

Financial disclosures: This study was supported by an unrestricted educational grant from Novartis Pharma B.V.

